# Species distribution and susceptibility profiles of Candida species isolated from vulvovaginal candidiasis, emergence of C. lusitaniae 

**DOI:** 10.18502/cmm.5.4.2062

**Published:** 2019

**Authors:** Seyed Ebrahim Hashemi, Tahereh Shokohi, Mahdi Abastabar, Narges Aslani, Mahbobeh Ghadamzadeh, Iman Haghani

**Affiliations:** 1 Student Research Committee, School of Medicine, Mazandaran University of Medical Sciences, Sari, Iran; 2 Invasive Fungi Research Centre (IFRC), Mazandaran University of Medical Sciences, Sari, Iran; 3 Department of Medical Mycology, School of Medicine, Mazandaran University of Medical Sciences, Sari, Iran; 4 Infectious and Tropical Diseases Research Center, Tabriz University of Medical Sciences, Tabriz, Iran; 5 Gynecology and Obstetrics Department of Hazrat-e- Zainab Hospital, Babolsar, Iran

**Keywords:** Antifungal susceptibility testing, C. lusitaniae, Candida species, PCR-RFLP, Vulvovaginal candidiasis

## Abstract

**Background and Purpose:**

The aim of the current study was to investigate the epidemiology of vulvovaginal candidiasis (VVC) and recurrent VVC (RVVC), as well as the antifungal susceptibility patterns of *Candida* species isolates.

**Materials and Methods:**

A cross-sectional study was carried out on 260 women suspected of VVC from February 2017 to January 2018. In order to identify *Candida* species isolated from the genital tracts, the isolates were subjected to polymerase chain reaction restriction fragment length polymorphism (PCR-RFLP) using enzymes *Msp I* and sequencing. Moreover, antifungal susceptibility testing was performed according to the Clinical and Laboratory Standards Institute guidelines (M27-A3).

**Results:**

Out of 250 subjects, 75 (28.8%) patients were affected by VVC, out of whom 15 (20%) cases had RVVC. Among the *Candida* species, *C. albicans* was the most common species (42/95; 44.21%), followed by *C. lusitaniae* (18/95; 18.95%), *C. parapsilosis* (13/95; 13.69%), *C. glabrata* (8/95; 8.42%), *C. kefyr* (6/95; 6.31%), *C. famata* (5/95; 5.26%), *C. africana *(2/95; 2.11%), and *C. orthopsilosis *(1/95; 1.05%), respectively. Multiple *Candida* species were observed in 28% (21/75) of the patients. Nystatin showed the narrowest range of minimum inhibitory concentration (MIC) (0.25-16 μg/ml) against all* Candida *strains, whereas fluconazole (0.063-64 μg/ml) demonstrated the widest MIC range. In the current study,* C. lusitaniae, *as the second most common causative agent of VVC, was susceptible to all antifungal agents. Furthermore, 61.1% of *C. lusitaniae *isolates were inhibited at a concentration of ≤ 2 μg/ml*, *while 38.9% (n=7) of them exhibited fluconazole MICs above the epidemiologic cutoff values (ECV). *Candida* species showed the highest overall resistance against fluconazole (61.3%), followed by itraconazole (45.2%) and caspofungin (23.7%). All of *C. albicans* strains were resistant to itraconazole with a MIC value of ≥ 1 μg/ml; in addition, 87.5% of them were resistant to fluconazole. Moreover, 100% and 87.5% of *C. glabrata* strains were resistant to caspofungin and fluconazole, respectively.

**Conclusion:**

As the findings revealed, the majority of VVC cases were caused by non-*albicans Candida* species which were often more resistant to antifungal agents. *Candida lusitaniae* generally had fluconazole MICs above the ECV. Given the propensity of *C. lusitaniae* to develop resistance under drug pressure, antifungals should be administered with caution. The emergence of these species justify the epidemiological surveillance surveys to watch out the distribution of yeast species.

## Introduction

Vulvovaginal candidiasis (VVC) is a kind of opportunistic fungal infection of the lower genital tract in females caused by different *Candida* species. This infection affects approximately 75% of child-bearing age women at least once in their lifetime and interferes with the quality of sex life. Based on the statistics, 5-10% of VVC patients suffer from recurrent VVC (RVVC) [1]. The VVC affects not only mature women but also young girls. The predisposing factors for young girls to develop VVC include the anatomical features of vagina and its closeness to the rectum, lumbar hair, small labia minor, thin vaginal epithelium, deficiency in estrogen hormones, and genetic issues [2, 3]. In addition, some of the risk factors associated with the development of VVC include antibacterial agent usage, hormonal fluctuations during pregnancy, immune system weakness, use of intrauterine devices for birth control, poor personal hygiene, metabolic disorders (e.g., diabetes mellitus), and stress [4, 5]. 

The VVC is a highly common problem in diabetic women, owing to a number of factors, such as increase of vaginal mucosa due to the high deposition of glycogen in vaginal tissue [6]. According to the results of the research conducted around the world, the most common causative agent of VVC is *Candida albicans* (77-95%), followed by non-*albicans Candida* (NAC) species (20-30%) [7-9]. However, the results of the recent studies are indicative of the growing increase of NAC species [10, 11]. Among NAC species, *C. lusitaniae* remains a less common cause of vulvovaginitis worldwide; however, this species has a noticeable role in the recurrence of vaginitis [1, 12]. Moreover, based on some reports, this species shows resistance to amphotericin B and cross-resistance to echinocandins and azoles [13, 14]. However, there are limited data regarding the antifungal susceptibility of *C. lusitaniae* causing VVC.

Azole antifungal agents can be used in the treatment of VVC like it is used for other superficial fungal infections [15]. It is difficult to perform an epidemiological investigation about the incidence, diagnosis, and treatment of VVC given the high rate of self-treatment with over-the-counter (OTC) medications and also the treatment of patients without prescribing laboratory examinations by physicians [16]. The incidence of VVC resistant to azole antifungal is on a growing trend due to the excessive administration of fluconazole and other azoles. The VVC self-diagnosis and self-treatment with OTC antifungal product can occasionally lead to the perpetuation of symptoms and RVVC [17]. 

Susceptibility to azoles in *Candida* species is highly variable. The treatment failure, as well as the recurrence and relapse of infection due to the emergence of NAC species resistant to conventional azoles is a worrisome problem [18, 19]. Regarding this, the present study was conducted to identify the etiologic agents of VVC among the women attending a Gynecology Clinic in Babolsar, northern Iran, using polymerase chain reaction restriction fragment length polymorphism (PCR-RFLP) and evaluation of the in vitro antifungal susceptibility of *Candida* species isolated from patients with VVC to eight antifungal drugs. These antifungals included fluconazole (FLC), itraconazole (ITC), miconazole, clotrimazole (CLO), nystatin (NYS), ketoconazole (KET), caspofungin (CAS), and tioconazole (TIC). The ultimate goal was to determine the role of multiple *Candida* species in the incidence of RVVC.

## Materials and Methods

***Fungal isolates and patient characterization***

A cross-sectional study was carried out on 260 non-pregnant women referring to the Gynecology and Obstetrics Department of Hazrat-e-Zainab Hospital, Babolsar, Iran, from February 2017 to January 2018. All participants enrolled in the study signed consent forms. The women with the clinical evidence of VVC, including burning, itching, cheesy discharge, and pain during intercourse were enrolled in the study. On the other hand, the subjects who had recent vagina douche or any form of antifungal therapy and those unwilling to participate were excluded from the study. The RVVC was defined as four or more episodes of culture-proved VVC in a year. 

Two samples of cervical/vaginal discharge were collected from each patient by means of sterile saline wetted cotton-tipped swabs. One swab was used for direct microscopy, and the other one was applied for culture assay. Preliminary diagnoses of specimens were performed using the KOH (10%) mount, gram stain, culture on Sabouraud dextrose agar (SDA) (Merck, Germany), SDA supplemented with 0.5% chloramphenicol, and CHROMagar *Candida* incubated at 37°C for 48-72 h [20]. The CHROMagar *Candida* as a differential culture medium can facilitate the identification of mix yeast species in the clinical sample presumptively. 

Serial dilutions of mix yeasts were set up on CHROMagar *Candida* to differentiate and recognition them. The evidence of budding yeast cell with pseudohyphae in direct microscopy and yeast growth was considered VVC. Species identification of grown yeast was performed conventionally using germ tube production in horse serum, chlamydospore test on corn meal agar with Tween 80, and colored colonies. For accurate identification, the yeast isolates were subjected to further investigation, including molecular methods.

***DNA Extraction***

Genomic DNA was extracted according to our previously described method [20] with some modifications. The isolates were identified by means of ITS1-5.8S-ITS2 gene amplification. Briefly, a loopful of 48-hour grown colonies was suspended in 300 µl of lysis buffer (200 mmol^-l ^Tris-HCl [pH: 7.5], 25 mmol^-l ^EDTA, 0.5% [w/v] SDS, 250 mmol^-l ^NaCl) and then incubated at 100°C for 15 min and centrifuged. The supernatant was added with 200 µl of 3.0 mol^–l^ sodium acetate and incubated at -20°C for an hour and then centrifuged at 12,000 g for 10 min. The supernatants were precipitated with an equal volume of cold isopropanol, centrifuged at 10,000 g for 10 min, washed with 70% of cold ethanol, air-dried, suspended in 50 µl TE buffer (10 mM Tris, 1 mM EDTA pH 8), and finally stored at -20°C until needed.

*Molecular identification*

For the purpose of molecular identification, the samples were subjected to ITS1-5.8S-ITS2 rDNA amplification and restriction enzyme analysis. The restriction enzyme analysis was performed as previously described [21]. Briefly, for each restriction digestion reaction, 10 μl of the amplified PCR product was digested with 1.5 μl of restriction enzyme buffer, 1 μl of restriction enzyme *Msp I* (Fisher Scientific, Leicestershire, UK), and 2.5 μl of high-performance liquid chromatography grade water. The reaction mixture (15 μl) was incubated at 37°C for 2 h. Restriction fragments were separated by 2% agarose gel in TBE buffer for 1 h at 100 V.

The identification of *C. albicans *species complex (i.e., *C. africana, C. albicans, *and* C. dubliniensis*) was accomplished using the partial amplification of hyphal wall proteins (HWP1) gene according to the primers designed by Romeo and Criseo (forward, 5′-GCTACCACTTCAGAATCATCATC-3′ and reverse 5′-GCACCTTCAGTCGTAGAGACG-3′) that generate the fragments of 940 and 740 bp for *C. albicans* and *C. africana* [22]. The discrimination of C. *parapsilosis* complex, including *C. parapsilosis *and *C. orthopsilosis,* was conducted as previously described [23].

***In vitro*** ***susceptibility testing***

Susceptibility of the grown yeasts to FLC, ITC, MIC, CLO, NYS, KET, CAS, and TIC was evaluated using broth microdilution method as recommended by the Clinical and Laboratory Standards Institute (CLSI) M27-A3 and M27-S4 document guidelines [24, 25]. Briefly, the antifungal agents were diluted in the standard RPMI-1640 medium (Sigma Chemical Co. Germany) buffered to pH 7.0 with 0.165 M-morpholinepropanesulfonic acid (Sigma, Germany) and L-glutamine without bicarbonate to yield two times their concentrations. 

The buffer medium was dispensed into 96-well microdilution trays at the concentrations of 0.016-16, 0.063-64, and 0.008-8 μg/ml for ITC/KET/NYS/TIC, FLC, and CAS, respectively. The MIC endpoint was defined as 100% and 80% inhibition for NYS and other drugs, respectively. Yeast inoculum onto Sabouraud dextrose in sterile saline (0.85%) was prepared after 24 h of incubation, resulting in a final concentration of 0.5-2.5×10^3^ cells/ml. The plates were incubated at 35°C for 48 h for all antifungals, except for CAS and FLC (for 24 h). For each isolate, drug-free (growth control) and yeast-free (drug control) wells were included, and all isolates were tested in duplicate. *Candida parapsilosis* (ATCC 22019) was used as a quality control for each series of MIC plate.

***Ethical approval***

Ethical approval was obtained from the Research and Ethics Committee of Mazandaran University of Medical Sciences, Mazandaran, Iran, with a reference number of IR.MAZUMS.REC.95.2313 and dated 22 September 2016.

## Results

***Isolation and identification of microorganisms***

A total of 260 women with the mean age of 32±9.8 years (age range: 17-51 years) suspected of *Candida* vaginitis were studied at Hazrat-e-Zainab Hospital, Babolsar, Northern of Iran. Based on the results, 75 (%28.8) patients were affected by VVC, out of whom 15 (20%) cases had RVVC. The mean ages of VVC and RVVC patients were 31±7.5 and 28 ±4.4 years, respectively, showing a significant difference (P=0.005). The most prevalent signs and symptoms were itching (32.7%), cheesy discharge (32.5%), burning (22.4%), and dyspareunia (12.3%) (Table 1). 

***Polymerase chain reaction restriction fragment length polymorphism and HWP1 amplification***

**Figure 1:**
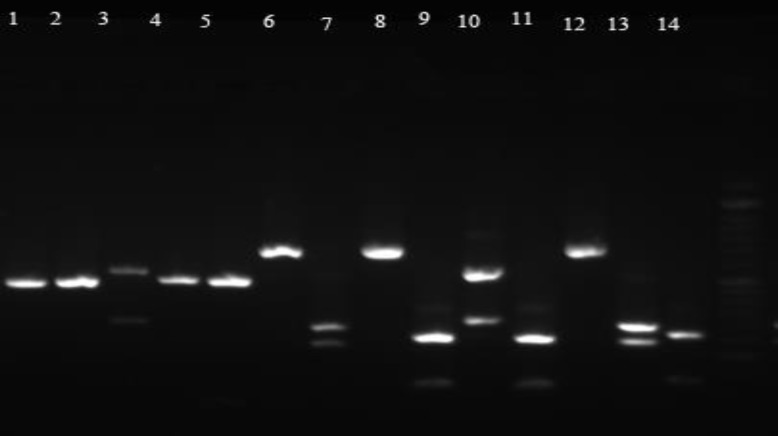
Polymerase chain reaction-restriction fragment length polymorphism assay (1.5% agarose gel electrophoresis) of ITS1-5.8S-ITS2 gene after restricting polymorphic region with *Msp*I enzyme; lanes 1, 2, 4, and 5) *C. parapsilosis*, lane 3) *C. glabrata*, lanes 6, 8, and 12) *C. famata*, lanes 7 and 13) *C. albicans*, lanes 9 and 11) *C. lusitaniae*, lane 10) *C. glabrata*, and last lane) a 100-bp molecular ladder

**Figure 2:**
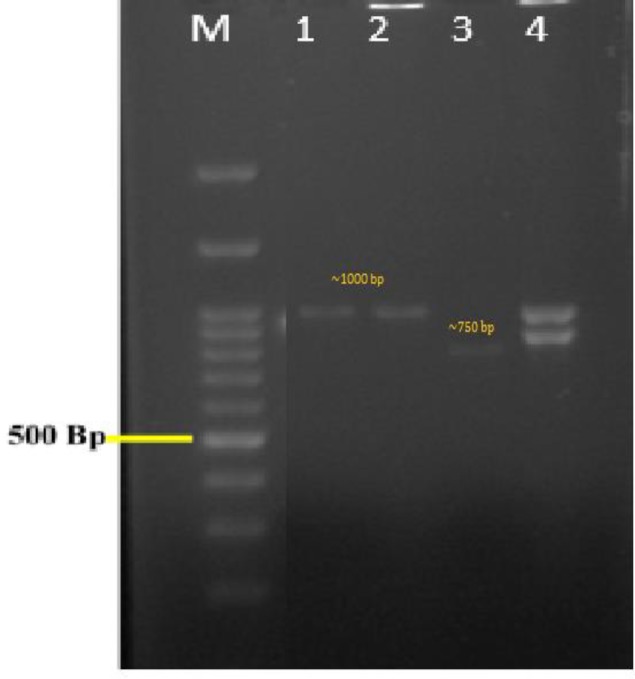
Polymerase chain reaction amplification of HWP1 gene; lanes 1, 2, and 4) *C. albicans*, lane 3) *C. africana*, and lane M) a 100-bp molecular ladder

Enzymatic digestion with *MspI* revealed different patterns for yeast isolates (Figure 1). Furthermore, the partial amplification of HWP1 gene for *C. albicans* and *C. africana* strains yielded a single band with sizes of ~1000 and 750 bp, respectively (Figure 2). A total of 95 *Candida* strains were isolated from 75 infected patients (Table 2). The most prevalent species was *C. albicans *(42/95; 44.22%), followed by *C. lusitaniae* (18/95; 18.95%), *C. parapsilosis* (13/95; 13.69%), *C. glabrata* (8/95; 8.41%), *C. kefyr* (6/95; 6.31%), *C. famata* (5/95; 5.26%), *C. africana* (2/95; 2.11%), and *C. orthopsilosis* (1/95; 1.05%) (Tables 2). Only one *Candida* species was identified in 72% (54/75) of the patients. Mixed infections with multiple *Candida* species (two or more) were observed in 28% (21/75) of the patients with *Candida* vulvovaginitis. Out of this group, 71.4% (15/21) of the patients suffered from RVVC. In 85.7% (18/21) of the patients with multiple species, *Candida albicans* were mixed with other NACs. In 19% (4/21) of the cases, 3 different *Candida* species were obtained from patients with RVVC (Table 3). Two *C. africana* isolates were mixed with other *Candida* species; therefore, it was not possible to determine their antifungal susceptibility due to the difficulty of separating them. 

**Table 1:** Signs and symptoms in patients with vulvovaginal candidiasis and recurrent vulvovaginal candidiasis regarding age groups

	**Age groups**										**Signs & symptoms**
**N (%)**	**RVVC** **N**					**VVC** **N**					
	**50-59**	**40-49**	**30-39**	**20-29**	**10-19**	**50-59**	**40-49**	**30-39**	**20-29**	**10-19**	
37 (49.3%)	-	-	3	7	-	1	4	8	14	-	Burning
53 (70.6%)	-	-	4	8	1	2	3	14	20	1	Itching
52 (69.3%)	-	-	4	8	1	1	3	13	20	2	Cheesy discharge
22 (29.3%)	-	-	1	5	1	1	2	5	6	1	Dyspareunia

**Table 2:** Identification of *Candida* species in patients with vulvovaginal candidiasis and recurrent vulvovaginal candidiasis regarding age groups using the amplification of ITS1‑ITS4 regions, restriction analysis, and partial amplification of hyphal wall proteins (HWP1) gene

**Total (%)**	**RVVC** **N (%)**					**VVC** **N (%)**					***Candida*** ** species**
	**50-59**	**40-49**	**30-39**	**20-29**	**10-19**	**50-59**	**40-49**	**30-39**	**20-29**	**10-19**	
42 (44.22)	-	-	3 (3.15)	8 (8.43)	1 (1.05)	-	4 (4.22)	12 (12.63)	14 (14.74)	-	*C. albicans*
18 (18.95)	-	-	-	3 (3.15)	-	1 (1.05)	2 (2.11)	3 (3.15)	7 (7.37)	2 (2.11)	*C. lusitaniae*
13 (13.69)	-	-	2 (2.11)	1 (1.05)	1 (1.05)	-	-	4 (4.22)	5 (5.26)	-	*C. parapsilosis*
8 (8.41)	-	-	2 (2.11)	3 (3.15)	-	1 (1.05)	-	1 (1.05)	1 (1.05)	-	*C. glabrata*
6 (6.31)	-	-	2 (2.11)	1 (1.05)	-	-	-	3 (3.15)	-	-	*C. kefyr*
5 (5.26)	-	-	-	2 (2.11)	-	1 (1.05)	1 (1.05)	-	1 (1.05)	*-*	*C. famata*
2 (2.11)			1 (1.05)		-	-	-	1 (1.05)	-		*C. africana*
1 (1.05)	-	-	-	1 (1.05)	-	-	-	-	-	-	*C. orthopsilosis*
95 (100)	-	-	10 (10.53)	19 (20)	2 (2.11)	3 (3.15)	7 (7.38)	24 (25.26)	28 (29.47)	2 (2.11)	Total (%)

**Table 3:** Distribution of multiple *Candida* species* in patients with recurrent vulvovaginal candidiasis and vulvovaginal candidiasis

***Candida *** **species**	**RVVC**	**VVC**	**Total**
*C. albicans and C. kefyr*	3	1	4
*C. glabrata and C. kefyr*	1	0	1
*C. albicans and C. parapsilosis*	2	0	2
*C. albicans and C. orthopsilosis*	1	0	1
*C. albicans and C. africana*	0	1	1
*C. albicans, C. parapsilosis, and C. lusitaniae*	1	0	1
*C. glabrata and C. famata*	1	0	1
*C. albicans and C. famata*	0	2	2
*C. albicans, C. kefyr, and C .lusitaniae*	1	0	1
*C. albicans, C. famata, and C. lusitaniae*	1	0	1
*C. albicans , C. glabrata, C. parapsilosis, and C. africana*	1	0	1
*C. albicans and C. lusitaniae*	1	2	3
*C. parapsilosis, C. glabrata, and C. lusitaniae*	1	0	1
*C. albicans and C. glabrata*	1	0	1
Total	15	6	21

**Table 4:** In vitro antifungal susceptibility of eight antifungal agents against 93 *Candida* species isolated from patients with vulvovaginal candidiasis (Numbers in red boldface are mode value)

**Isolates**	**Antifungal agents**	**0.016**	**0.032**	**0.063**	**0.125**	**0.25**	**0.5**	**1**	**2**	**4**	**8**	**16**	**32**	**64**	**%Resistant (n)**	**%I(n)**	**%S(n)**	**MIC Range** **µg/mL**	**MIC50 µg/mL**	**MIC90** **µg/mL**	**G-Mean** **µg/mL**	**Mode** **µg/mL**
*C. albicans* (n=42)	FLC									6	7		3	26	85.7% (36)	14.3% (6)	0	4-64	64	64	28.5	64
	ITC								3	6	10	22	1		100% (42)	0	0	2-32	16	16	8.8	16
	MIC						2	3	6	21	5	5			-	-	-	0.5-16	4	15.2	3.80	4
	CLO							1	11	1	4	25			-	-	-	1-16	16	16	7.48	16
	NYS					2	6	13	16	5					-	-	-	0.25-4	1.5	3.8	1.30	2
	KET						2	1	1	11	7	20			-	-	-	0.5-16	8	16	7.48	16
	CAS			13		7	4	12	1	5					42.8% (18)	9.5% (4)	%47.7(20)	0.063-4	0.5	3.8	0.37	0.063
	TIC							2	3	11	6	20			-	-	-	1-16	8	16	7.61	16
*C. lusitaniae *(n=18)	FLC						2		9	1	1	2		3	-	-	-	0.5-64	2	16	3.26	2
	ITC						2	11	4	1						-	-	0.5-4	1	2	1.27	1
	MIC					1	9	4	2	2					-	-	-	0.25-4	0.5	2.8	0.78	0.5
	CLO							12	6						-	-	-	1-2	1	2	1.44	1
	NYS						2	12	4						-	-	-	0.5-2	1	2	1.08	1
	KET					1	12	4			1				-	-	-	0.25-8	0.5	1	0.66	0.5
	CAS					10	4	4							-	-	-	0.25-1	0.25	1	0.40	0.25
	TIC						13	2	3						-	-	-	0.5-2	0.5	1.4	0.61	0.5
*C. parapsilosis* (n=13)	FLC								5	1	1	2		4	57.1% (8)	7.1% (1)	%35.7(5)	2-64	12	64	10.76	2
	ITC						1	1	5		1	5			-	-	-	0.5-16	5	16	4.64	16
	MIC							4	3	1	1	4			-	-	-	1-16	3	16	4	16
	CLO								2	5	1	5			-	-	-	2-16	6	16	6.89	4
	NYS						1			7		5			-	-	-	0.5-16	4	16	6.24	4
	KET				1	2	4					6			-	-	-	0. 125-16	8.25	16	2.002	16
	CAS			1					2	5	5				42.8% (6)	35.7% (5)	%21.4(3)	0.032-8	4	8	3.45	8
	TIC			1						2	7	3			-	-	-	0.063-8	8	16	6.24	8
*C. glabrata* (n=8)	FLC										1			7	87.5% (7)	12.5% (1)		8-64	64	64	45.25	64
	ITC							1			4	3			-	-	-	1-16	8	16	8	8
	MIC								1		5	2			-	-	-	2-16	8	16	8	8
	CLO						1								-	-	-	0.5-16	16	16	10.37	16
	NYS						1	2				5			-	-	-	0.5-16	16	16	5.18	16
	KET							5				3			-	-	-	1-16	1	16	2.82	1
	CAS						2	1	2	1	2				100% (8)	0	0	0.5-8	2	8	2	0.5
	TIC					2		1	2	1	1	1			-	-	-	0.25-16	2	10.4	1.83	0.25
*C. kefyr* *(n=6)*	FLC												2	4	100% (6)	0	0	32-64	64	64	50.79	64
	ITC									2	2	2			-	-	-	4-16	8	16	8	4
	MIC							1			1	4			-	-	-	1-16	16	16	8.97	16
	CLO										1	5			-	-	-	8-16	16	16	14.25	16
	NYS							1	3		2				-	-	-	1-8	2	8	2.82	2
	KET										6				-	-	-	8-8	8	8	8	8
	CAS					1			5						100% (6)	0		0. 25-2	2	2	1.41	2
	TIC				2		1			1	2				-	-	-	0.125-4	2.25	8	1.12	0.125
*C. famata* (n=5)	FLC			3					1		1				-	-	-	0.063-8	0.063	5.6	0.33	0.063
	ITC	1	2					1			1				-	-	-	0.016-8	0.032	5.2	0.167	0.032
	MIC				4			1							-	-	-	0.125-1	0.125	0.65	0.189	0.125
	CLO				3		1				1				-	-	-	0.125-8	0.125	5	0.37	0.125
	NYS					3	1	1							-	-	-	0.25-1	0.25	0.8	0.37	0.25
	KET	2	1				1		1						-	-	-	0.016-2	0.032	1.4	0.096	0.016
	CAS			1	2	1	1								-	-	-	0.063-0.5	0.125	0.4	0.162	0.125
	TIC	1	1	1		1	1								-	-	-	0.016-0.5	0.063	0.4	0.083	0.016
*C. orthopsilosis* (n=1)	FLC													1	100% (1)			ND	ND	ND	ND	-
	ITC											1			-	-	-	ND	ND	ND	ND	-
	MIC											1			-	-	-	ND	ND	ND	ND	-
	CLO											1			-	-	-	ND	ND	ND	ND	-
	NYS											1			-	-	-	ND	ND	ND	ND	-
	KET											1			-	-	-	ND	ND	ND	ND	-
	CAS										1				100% (1)			ND	ND	ND	ND	-
	TIC											1						ND	ND	ND	ND	-
*All Candida *species (n = 93)	FLC			3			2		15	8	11	4	5	45	61.3% (57)	8	5	0.063-64	32	64	13.68	64
	ITC	1	2				3	14	12	9	18	33	1		45.2% (42)	-	-	0.016-32	8	16	4.37	16
	MIC				4	1	11	13	12	24	12	16						0.125-16	4	16	2.69	4
	CLO				3		2	13	19	6	7	43			-	-	-	0.125-16	8	16	4.89	16
	NYS					5	11	29	23	12	2	11			-	-	-	0.25-16	2	16	1.76	1
	KET	2	1		1	3	19	10	2	11	14	30			-	-	-	0.016-16	4	16	2.77	16
	CAS			15	2	19	11	17	10	11	8				23.7% (22)	9	29	0.063-8	0.5	4	0.64	0.25
	TIC	1	1	2	2	3	15	5	8	15	16	25			-	-	-	0.016-16	4	16	2.77	16

FLC: fluconazole, ITC: itraconazole, MIC: miconazole, CLO: clotrimazole, NYS: nystatin, KET: ketoconazole, CAS: caspofungin, TIC: tioconazole, ND: not determined, R: resistance, I: intermediate, S: susceptible

***In vitro*** ***susceptibility testing ***

Table 4 summarizes the MIC ranges, MIC_50_, MIC_90_, and geometric mean (GM) MIC values of antifungal drugs against all *Candida* isolates. The widest MIC range for all *Candida* strains was obtained for FLC (0.063-64 μg/ml), while the narrowest MIC range found for NYS (0.25-16 μg/ml). The GM MIC values for CAS, NYS, MIC, KET/TIC, ITC, CLO, and FLC against all strains were 0.64, 1.76, 2.69, 2.77, 4.37, 4.89, and 13.68 µg/mL, respectively. *Candida albicans*, isolated in this study, demonstrated greatest resistance to FLC (n=36; 85.7%); in this regard, the growth of only 6 (14.3%) isolates were inhibited at ≤ 4 μg/ml (Table 4). Moreover, all *C. albicans* strains were resistant to ITC with a MIC value of ≥ 1 μg/ml.

In the current study, *C. lusitaniae*, as the second most common causative agent of VVC, showed susceptibility to all antifungals. In addition, the growth of the majority of these species was inhibited at a concentration of ≤ 2 μg/ml (Table 4). Seven *C. lusitaniae* isolates exhibited FLC MICs above the epidemiologic cutoff values (ECV; 4-64 μg/ml). Six isolates of *C. kefyr* showed the highest susceptibility to CAS and TIC with the GM MIC values of 1.41 and 1.12 μg/ml, respectively (Table 4). Furthermore, CAS and TIC also inhibited the growth of 5 *C. famata* isolates at a concentration of ≤ 0.5 μg/ml. All of *C. glabrata* strains were resistant to CAS, and 87.5% of them were resistant to FLC (Table 4).

## Discussion

Vulvovaginal candidiasis is a common lower genital tract infection in pregnant and child bearing age women. The majority of patients with VVC are diagnosed by signs and symptoms without using laboratory findings, and infection is not confirmed. The present study targeted non-pregnant women in order to identify the distribution of *Candida* species in VVC and RVVC cases and determine their antifungal susceptibility patterns. In this study, the prevalence of VVC and RVVC was estimated by laboratory and clinical criteria. Although the prevalence rate of VVC (28.8%) in our study was within the reported range, it was slightly higher than the rates reported by Abbasi Nejat et al. [26], Diba et al. [27], and Hedayati et al. [28]. However, our obtained rate was lower than the prevalence rates presented by Mukasa et al. [29] and Bitew et al. [30]. 

Some of the potential factors for differences in the occurrence and/or recurrence of VVC among studies are sociodemographic characteristics, diabetes mellitus, dietary habits, personal hygiene, sexual activity, immunological status, and use of antibiotics, immunosuppressant, or oral contraceptives, which are various and conflicting [30]. In the current study, age was investigated as a possible risk factor for the occurrence of VVC and RVVC. Our finding regarding age as an important risk factor was consistent with those of similar earlier studies [27, 28] (Table 1). In the current study, the mean ages of the patients with VVC and RVVC were 31±7.5 and 28±4.4 years, respectively, showing a significant difference (P=0.005). Our finding is consistent with those obtained by Bitew et al. [30]. 

Furthermore, in the current study, out of 75 patients with VVC, 15 (20%) patients were diagnosed with RVVC. The prevalence rates of RVVC were reported as 24.2% and 12.2% in previous studies [26, 27]. In the current investigation, a total of six *Candida* species were detected. The prevalence rate of *C. albicans* as the most prevalent species associated with vulvovaginitis was obtained as 44.22%. Furthermore, the overall prevalence of NAC species was 55.78% with *C. lusitaniae* as the most predominant species. 

The NAC species isolated from the patients complaining of genital tract infection included *C. lusitaniae* (n=18, 18.95%), *C. parapsilosis* (n=13, 13.69%), *C. glabrata* (n=8, 8.41%), *C. kefyr* (n=6, 6.31 %), *C. famata* (n=5, 5.26%), C*. africana* (n=2, 2.11%), and *C. orthopsilosis* (n=1, 1.05%). In our study, *C. lusitaniae* was the second most prevalent isolate. *Candida lusitaniae* is an opportunistic yeast isolated much less commonly than other *Candida *species causing vaginitis. It was first described as a common isolate from the gastrointestinal tract of warm-blool animals [31]. 

*Candida lusitaniae* is an emerging yeast pathogen that infects immunocompromised patients with cancer and HIV/AIDS (32-38). This species has been isolated from the urine, bronchoalveolar lavage fluid, blood, peritoneal fluid, kidney, vagina, and skin [38-42]. *Candida lusitaniae* has been rarely recovered (0.6-2.5%) from patients with candidemia [43, 44]. It is haploid and germ tube negative and like *C. glabrata* has propensity to develop resistance to antifungals mainly to amphotericin B, azoles, and fluocytosine [45]. In CHROMagar *Candida* medium (CHROMagar Company, Paris, France), light to dark brown colored colonies are grown. This species is phylogenetically related to *C. auris* [46]. 

In a study, infection with *C*. *lusitaniae *showed a high rate of intrinsic resistance to amphotericin so that susceptibility testing was not required [47]. Although *C. lusitaniae* clinical isolates show no reduced susceptibility [44], in some research, it elevated the MICs for echinocandins [48], which are used as the first-line therapy of candidemia. Recently, an unusual emerging resistance to echinocandins has been shown due to mutation in *FKS* genes in clinical cases and experimental animal model [49]. In addition, there is evidence regarding the development of cross-resistance to azoles and echinocandins following combination antifungal treatment [14]. 

In our study, caspofungin values were below the ECVs of 0.5-1 μg/ml that would be considered non-wild using the ECVs reported by Lockhart et al. [48]. There is no valid CLSI susceptibility breakpoint for less prevalent *Candida *species like *C. lusitaniae*. In this case, the ECVs defined as the highest susceptibility endpoint of the wild-type MIC population. The ECVs can facilitate the detection of the emergence of in vitro resistance and help physicians in managing fungal infection where breakpoints are not available [50]. However, these values will not categorize a fungal isolate as susceptible or resistant and do not predict clinical response as breakpoints do [51] 

The recovery rate of *C. albicans* as the most common species isolated from patients with VVC was similar with those of numerous studies. Nonetheless, the recovery percentage of NAC vaginitis (55.78%) was higher than the rates reported in two previous studies presenting lower rates of 28.7% and 41.4%, respectively [25, 29]. Similar to other studies that reported a high recovery percentage (65.0% and 57.5%) for NAC species in Egypt and Iran [28, 52], it seems that there has been a growing shift towards NAC species.

Documented information regarding the prevalence of NAC isolated from Iranian patients causing vulvovaginitis revealed *C. glabrata* as the most common yeast among the NAC species causing vaginitis [26, 28, 53]. It should be noted that *C. lusitaniae *has been rarely reported as the causative agent of VVC in the studies conducted in Iran. It seems that the high prevalence of *C. lusitaniae* in Babolsar, Iran, is related to some factors, such as the accurate identification of this agent from other *Candida* species using PCR-RFLP and sequencing, quite good sampling, genetic adaptability of this species to this geographic area, and different populations. In addition, there is no report regarding the distribution pattern of *Candida* species in this region. 

In a study performed by Mukasa et al. [29], *C. glabrata* was reported as the most commonly isolated NAC species (14.3%), followed by *C. krusei *(3.3%), *C. parapsilosis* (8.9%), *C. tropicalis* (1.44%), *C. famata* (0.96%), *C. parapsilosis* (0.48%), and *C. lusitaniae* (0.48%). In contrast to our study, *C. lusitaniae* was the dominant NAC species (18.95%), followed by *C. parapsilosis* (13.69%), *C. glabrata* (8.41%), *C. kefyr* (6.5%), and *C. famata* (6.31%). 

Similar to our study, in a study carried out by Bitew et al. [30], different recovery rates were reported for NAC species. The results of the mentioned study demonstrated *C. krusei* as the dominant NAC species, followed by *C. dubliniensis*, *C. glabrata*, *C. tropicalis*, *C. kefyr*, *C. parapsilosis*, *C. guilliermondii*, *C. lusitaniae,* and *C. inconspicua.* It was suggested that the increase of NAC species, isolated from patients complaining of genital tract infection naturally resistant to antifungal agents, is probably related to the widespread and inappropriate use of azole antifungals [1, 54]. 

Therefore, the most logical cause of NAC species emergence is low sensitivity to azole antifungal agents, compared with *Candida* species isolated frequently from patients [55]. Similar to other studies, mixed infection was seen in our patients [1]. Laboratories should be able to detect mixed cultures in primary cultures because it is an important issue for the management of VVC patients. In addition, the determination of in vitro antifungal susceptibility pattern is highly important before deciding on a specific treatment and introducing new antifungal agents in order to predict the outcome of treatment for the routine surveillance of fungal infections. 

Fluconazole is first-line therapy for the treatment and prevention of candidiasis; however, the prolonged use of this antifungal agent has contributed to the development of antifungal drug resistance in *Candida* isolates. In the present study, *C. kefyr*, *C. glabrata/C. albicans, *and *C. parapsilosis* showed the resistance rates of 100%, 87%, and 57% to FLC, respectively, which were higher, compared to the values reported in other studies [1, 26, 29]. Regarding ITC, *C. glabrata *was absolutely resistant to this medication, which is similar to the results obtained by Mukasa et al. [29]. 

Unlike other reports, in the current study, *C. albicans* and *C. parapsilosis* were found to be absolutely resistant to ITC. Abbasi Nejat et al. [26] found that all isolates were highly susceptible to amphotericin B. With regard to CAS, the resistance rates were obtained as 100% and 42.8% for *C. glabrata *and *C. albicans*/*C. parapsilosis, *respectively*.* In contrast, Bitew et al. [30] found that all of the yeast isolates were 100% susceptible to this medication. In the present study, the isolates showed reduced MICs to caspofungin with an MIC_90_ of 8 μg/ml and resistance rate of 23.7%. 

Overall, in terms of GM MICs, CAS demonstrated potent activity against almost all yeast isolates (n=63) in comparison with FLC, ITC, MIC, CLO, NYS, KET, and TIC. In this study, *C. famata* was susceptible to all medications. While in a study performed in Uganda, 100% of *C. famata* showed resistance to ITC, and 50% of them were resistant to CLO [29]. The high prevalence rate of NAC species with reduced susceptibility to azole antifungal agent in the current study is in line with some recent reports that have indicated that resistance to antifungal drugs may also be an important factor for VVC.

## Conclusion

As the findings indicated, NAC species were the most common yeast isolates obtained from patients with VVC infection and *C. lusitaniae* being the generally predominant species. Given the propensity of *C. lusitaniae* to develop resistance under drug pressure, antifungals should be administered with caution. The emergence of these species justifies the implementation of epidemiological surveillance surveys to watch out the distribution of yeast species. Overall, in vitro antifungal susceptibility testing is an essential measure for choosing the correct antifungal agents for appropriate therapy. However, it is required to perform prospective studies in our region to track the changing trend in antifungal susceptibility and development of mutation under the widespread use and abuse of OTC antifungals, especially in RVVC cases.
